# Changes in bacterial community composition of *Escherichia coli* O157:H7 super-shedder cattle occur in the lower intestine

**DOI:** 10.1371/journal.pone.0170050

**Published:** 2017-01-31

**Authors:** Rahat Zaheer, Eric Dugat-Bony, Devon Holman, Elodie Cousteix, Yong Xu, Krysty Munns, Lorna J. Selinger, Rutn Barbieri, Trevor Alexander, Tim A. McAllister, L. Brent Selinger

**Affiliations:** 1 Agriculture and Agri-Food Canada, Lethbridge Research and Development Centre, Lethbridge, Alberta, Canada; 2 UMR Génie et Microbiologie des Procédés Alimentaires, AgroParisTech, INRA, Université Paris-Saclay, Thiverval-Grignon, France; 3 Department of Biological Sciences, University of Lethbridge, Lethbridge, Alberta, Canada; Wageningen University, NETHERLANDS

## Abstract

*Escherichia coli* O157:H7 is a foodborne pathogen that colonizes ruminants. Cattle are considered the primary reservoir of *E*. *coli* O157:H7 with super-shedders, defined as individuals excreting > 10^4^ *E*. *coli* O157:H7 CFU g^-1^ feces. The mechanisms leading to the super-shedding condition are largely unknown. Here, we used 16S rRNA gene pyrosequencing to examine the composition of the fecal bacterial community in order to investigate changes in the bacterial microbiota at several locations along the digestive tract (from the duodenum to the rectal-anal junction) in 5 steers previously identified as super-shedders and 5 non-shedders. The overall bacterial community structure did not differ by *E*. *coli* O157:H7 shedding status; but several differences in the relative abundance of taxa and OTUs were noted between the two groups. The genus *Prevotella* was most enriched in the non-shedders while the genus *Ruminococcus* and the *Bacteroidetes* phylum were notably enriched in the super-shedders. There was greater bacterial diversity and richness in samples collected from the lower- as compared to the upper gastrointestinal tract (GI). The spiral colon was the only GI location that differed in terms of bacterial diversity between super-shedders and non-shedders. These findings reinforced linkages between *E*. *coli* O157:H7 colonization in cattle and the nature of the microbial community inhabiting the digestive tract of super-shedders.

## Introduction

*Escherichia coli* O157:H7 is the serotype responsible for the majority of human enterohemorrhagic *E*. *coli* (EHEC) infections in most industrial countries [1]. Cattle are considered the primary reservoir of this pathogen although other animals may be carriers [2]. A number of epidemiological studies have shown that up to 30% of feedlot cattle shed *E*. *coli* O157:H7 in their feces [3, 4, 5, 6]. Due to public health concerns and the role of cattle in disseminating *E*. *coli* O157:H7, this microorganism has a significant negative impact on the beef production industry.

Heterogeneous shedding among cattle has been observed, where most animals shed only transiently low levels (< 100 CFU g^-1^ feces) of *E*. *coli* O157:H7 while a small number of cattle excrete large quantities of this pathogen or shed lower levels over longer periods of time [7]. The term ‘super-shedder’ was first introduced by Matthews *et al*., [8, 9] and defined by Chase-Topping *et al*., [10] in 2008 as an animal that excretes >10^4^ CFU of *E*. *coli* O157:H7 per g of feces. Despite their low prevalence within a feedlot (typically <1% of animals), super-shedders are believed to be responsible for the majority of *E*. *coli* O157:H7 shed in the feces of cattle and the largest transmitters of this pathogen in feedlots [8, 9, 11].

The primary site of *E*. *coli* O157:H7 colonization in cattle has been shown to be the rectal-anal junction (RAJ) [12, 13] and strong associations between RAJ colonization and super-shedder and/or long-term shedder status have been established [14, 15]. The gallbladder has also been suggested as a possible niche for *E*. *coli* O157:H7 in cattle [16]. Since cattle are generally asymptomatic carriers of *E*. *coli* O157:H7, this bacterium has been described as a commensal. However, the fact that *E*. *coli* O157:H7 can cause intestinal inflammation and induce small mucosal haemorrhages and focal petechiae in the cattle intestine as well as induce innate and adaptive immune responses suggests that it could also be viewed as an opportunistic bovine pathogen [17]. Nonetheless, little is known about the etiology of this infection in cattle. Some factors, such as diet, the presence of specific bacteriophages, and other pathogenic strains have been shown to be associated with *E*. *coli* O157:H7 infection in cattle [18, 19, 20, 21]. However, these factors alone do not explain the mechanisms that lead to the development of super-shedders and the differences in carriage status among animals.

In a recent study by our group using 16S rRNA gene pyrosequencing, the fecal bacterial microbiota of *E*. *coli* O157:H7 super-shedders differed significantly from that of non-shedders originating from a feedlot in southern Alberta [22]. This finding supports the idea that microbiota composition may play a critical role in the establishment and/or ecology of *E*. *coli* O157:H7 within the intestinal tract of cattle. In the present study, we used 16S rRNA gene pyrosequencing to examine changes in bacterial diversity and community structure along the small and large intestine of cattle identified as super-shedders and non-shedders. The present study is aimed at comparing the composition and structure of the gastrointestinal (GI) microbiota at several different GI locations in *E*. *coli* O157:H7 super-shedders and non-shedders to determine if previously observed differences in fecal communities were associated with specific regions of the intestinal tract.

## Materials and methods

### Super-shedder identification and study design

The identification of *E*. *coli* O157:H7 super-shedders within the feedlot has been previously described in detail [22, 23]. Briefly, fecal samples were collected from 400 crossbred Charolaise yearling feedlot beef steers with body weights ranging between 500–525 Kg and housed at a single feedlot (Lethbridge, Alberta, Canada). All cattle were fed a barley-grain based finishing diet. *E*. *coli* O157:H7 super-shedders (>10^4^ *E*. *coli* O157:H7 CFU/g of fecal material) were identified by enumeration on CT-SMAC (Sorbitol MacConkey with Tellurite and Cefixime; Dalynn Biologicals, Calgary, Alberta, Canada) as described by Niu *et al*., [20]. Three representative non-sorbitol fermenting colonies from each sampling point were confirmed to be *E*. *coli* O157 using the *E*. *coli* O157 latex test kit (Oxoid Ltd., Basingstoke, Hampshire, UK). Positive agglutination isolates were confirmed by multiplex PCR to test for the presence of genes specific to the O157:H7 serotype [24]. When *E*. *coli* was not detectable by plating, duplicate 1 g subsamples of feces were enriched in 9 mL of modified TSB containing novobiocin (20 mg/L; Sigma-Aldrich Canada Co., Oakville, ON, Canada), bile salts (1.5 g/L; BD—Canada, Mississauga, ON, Canada), dipotassium phosphate (1.5 g/L; Sigma-Aldrich Canada Co.) and TSB (30 g/L; BD—Canada) and incubated for 6 h at 37°C. Enriched samples were then subject to immunomagnetic separation (IMS) using anti-*E*. *coli* O157:H7 Dynabeads (Invitrogen, Carlsbad, CA) as per manufacturer's instructions. A 50 μL aliquot of bead-bacteria complex was plated onto CT-SMAC (Dalynn Biologicals) and incubated at 37°C for 18 to 24 h. Three non-sorbitol fermenting clear colonies were randomly selected for latex-test and PCR confirmation as described above. Cattle that were negative both by enumeration and IMS were classified as non-shedders.

Five super-shedders (>10^4^ *E*. *coli* O157:H7 CFU/g of feces) from two pens and five non-shedders (negative for IMS) contemporary pen mates were purchased and transported to the Lethbridge Research and Development Centre (LeRDC). Steers were kept on the same barley-grain based finishing diet as in the feedlot. The shedding pattern of all steers was monitored daily until the date of slaughter as described above. Steers were slaughtered over a period of 6 days following the abattoir’s schedule with 2 animals (1 shedder + 1 non-shedder) slaughtered on the next day, 4 animals (2 shedder + 2 non-shedder) on the 4^th^ day and 4 animals (2 shedder + 2 non-shedder) on the 6th day of original identification and arrival at LeRDC. A typical captive bolt procedure according to the animal humane handling and slaughter guidelines by the Canadian Food Inspection Agency was used for pre-slaughter stunning by experienced abattoir staff immediately followed by the jugular vein bleeding. Steers were sequentially slaughtered as it was not possible to slaughter and conduct the necessary intestinal dissections from all cattle within a single day. This study was carried out in strict accordance with the recommendations and guidance of the Canadian Council of Animal Care. The protocol was reviewed and approved by the Lethbridge Research Centre Animal Care Committee.

### Digesta and tissue sample collection

Within 10 min of slaughter, the gastrointestinal tract (GIT) of each animal was removed and placed on a clean sheet of plastic on the floor of the abattoir. Eight intestinal sections, approximately 20 cm in length, were collected and aseptically removed from each animal from the following 8 locations in anatomical order: duodenum, proximal jejunum, mid jejunum, distal jejunum, cecum, spiral colon, descending colon and rectum at the RAJ. Bilateral ligatures were applied adjacent to the excision sites to minimize external contamination of the tissues with the digesta. During the excision, the work areas and tools were decontaminated with 70% ethanol and rinsed with sterile water to prevent cross-contamination among samples. Longitudinal incisions were made in each ligated GIT section and the solid and/or semisolid digesta were gently removed (quantities strongly varied between the different GIT sections and between animals), collected in 50 ml falcon tubes, immediately flash frozen in liquid nitrogen and stored at -80°C. After a gentle wash with sterile water, small pieces of the rectum tissue at the RAJ were scraped using a spatula and the samples were collected in 15 ml falcon tubes, immediately flash frozen in liquid nitrogen and stored at -80°C. Samples of the distal ileum were not collected as this tissue was designated as “specified risk material” and could not be legally removed from the abattoir.

### Metagenomic DNA extraction

Metagenomic DNA was extracted from each digesta and tissue sample using the Repeated Bead Beating + Column protocol (RBB+C), originally described by Yu and Morrison [25]. Briefly, 0.25 g of each sample was subjected to two rounds of bead-beating in a SDS containing buffer with 15 min of heating at 70°C. DNA was then precipitated with isopropanol, treated with RNase A (Qiagen), and purified using the column provided in the QIAmp DNA Stool Mini Kit (Qiagen). DNA samples were quantified using a NanoDrop 2000 spectrophotometer (Thermo Scientific).

### Pyrosequencing of the 16S rRNA gene

The 16S rRNA gene in each extracted DNA sample was amplified and sequenced at Molecular Research LP (Shallowater, Texas, USA) using bacterial tag-encoded FLX amplicon pyrosequencing (bTEFAP), as originally described by Dowd *et al*., [26]. Briefly, the HotStarTaq Plus Master Mix Kit, together with primers 27F (5’-GAGTTTGATCNTGGCTCAG-3’) and 519R (5’-GTNTTACNGCGGCKGCTG-3’), was used to amplify the hypervariable regions V1 to V3 of the 16S rRNA gene. The PCR program used to generate these amplicons consisted of: initial denaturation at 94°C for 3 min, followed by 28 cycles of 94°C for 30 s, 53°C for 40 s and 72°C for 1 min, with a final extension step at 72°C for 5 min. To enable multiplexing of samples, each sample was indexed with a unique 8-bp barcode. PCR products from all samples were then mixed in equal concentrations and purified using Agencourt Ampure beads (Agencourt Bioscience Corporation, MA, USA). Sequencing of the 16S rRNA gene amplicons was then carried out using a Roche 454 FLX Titanium system (San Francisco, CA, USA) following manufacturer’s instructions.

### 16S rRNA gene sequencing analysis

All 16S rRNA gene sequences were processed and analyzed within the QIIME software package v. 1.9.1 [27]. Reads were first demultiplexed with the removal of barcodes and primer sequences. Next, sequences were trimmed and quality filtered with the removal of sequences that were: > 550 bp, < 250, had a Phred score of < 30, or had homopolymer runs of > 6 bp. Chimeric sequences were identified and removed using the UCHIME algorithm [28] implemented in USEARCH v. 6.1544 [29]. The chimera-free sequences were then clustered into OTUs (operational taxonomic units) at 97% similarity using the open reference OTU picking protocol in QIIME and the Greengenes 13_5 database [30]. Sequences that did not match OTUs in the Greengenes database were clustered into OTUs using the *de novo* approach and USEARCH. The UCLUST consensus taxonomy assigner [29] was used to assign taxonomy to OTUs using Greengenes, with a minimum similarity of 0.9 and max accepts of 3. Representative sequences for the OTUs were aligned using PyNast [31]. Singletons, that is those OTUs found only once in the entire dataset, were also removed prior to analysis.

To account for uneven sequencing depths among samples, all samples were randomly subsampled to 3,100 sequences. This necessitated the loss of five samples from the analysis: one from the rectum and two each from the distal jejunum and cecum. The diversity within each sample (alpha diversity) was calculated within QIIME using the Chao1 estimator [32], observed OTUs, Shannon index [33], and PD whole tree [34] metrics. Diversity metrics for each intestinal section and shedding status group were analyzed using a two-way ANOVA implemented in PROC MIXED in SAS (version 9.4; SAS Institute Inc., Cary, NC, USA) followed by pairwise comparisons using Tukey’s HSD (honest significant difference; P < 0.05). The bacterial community structure (beta diversity) was evaluated using weighted UniFrac distances [35] and visualized using PCoA and the program EMPeror [36]. ANOSIM (analysis of similarities) with 999 permutations was used to compare the weighted UniFrac distances. OTUs that were differentially abundant among the eight different intestinal sections as well as between super-shedders and non-shedders were identified using G-test together with the Benjamini-Hochberg false discovery rate (FDR) correction [37] for multiple comparisons (FDR < 0.05).

Linear discriminant analysis effect size (LEfSe) was used to determine which taxa groups differed according to *E*.*coli* O157:H7 shedding status and GI section. LEfSe includes the Kruskal-Wallis test to identify different (P < 0.05) taxa among groups of samples followed by linear discriminant analysis which estimates the size of each of these differences [38].

## Results

### *Escherichia coli* O157:H7 shedding pattern at the date of slaughter

At the beginning of the experiment, 5 super-shedders (> 10^4^ CFU/g of feces detected by direct plating on CT-SMAC) and 5 non-shedders (no detection neither by direct plating on CT-SMAC nor by enrichment using immuno-magnetic separation) were identified. The shedding pattern of the five super-shedders was followed twice daily until slaughter as described by Munns *et al*., [23]. Only one steer, ID 310, met the definition of a super-shedder at the time of slaughter. However, *E*. *coli* O157:H7 was consistently isolated in fecal samples until slaughter from all steers originally identified as super-shedders. Remaining steers were slaughtered 4 (IDs 274 and 287) and 6 days (IDs 294 and 299) after arrival at LeRDC. *E*. *coli* O157:H7 was not detected in the five non-shedders during the study or at slaughter. The non-shedders were slaughtered along with the shedders on days 2 (ID 165), 4 (IDs 108 and 745) and 6 (IDs 152 and 242).

### Bovine gastrointestinal tract microbiota

**Fig 1:**
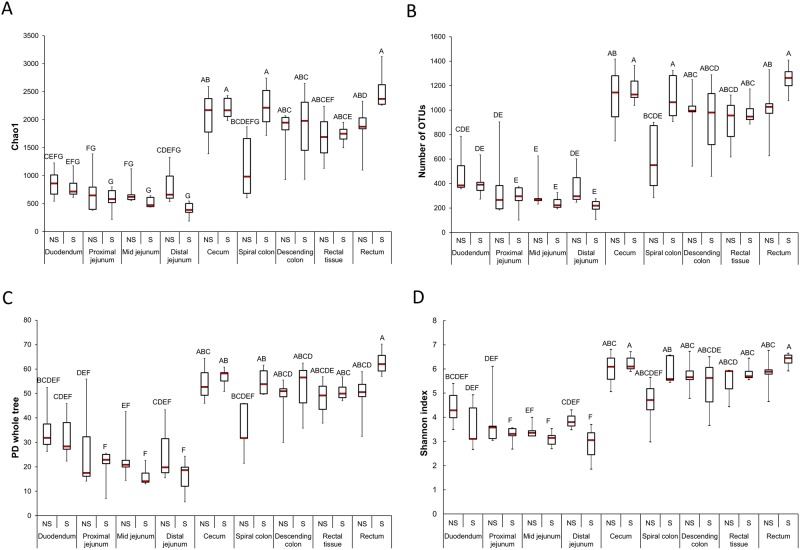
Box plots displaying the bacterial diversity and richness for *E*. *coli* O157:H7 shedder (S) and non-shedder (NS) cattle at each gastrointestinal section (n = 10). A) Chao1, B) number of OTUs, C) PD whole tree, and D) Shannon index (median ± interquartile range).

Eighty-four 16S rRNA gene libraries, corresponding to the 84 digesta and tissue samples, were sequenced using bacterial tag-encoded FLX amplicon pyrosequencing (bTEFAP). A total of 755,891 sequences with an average length of 458 bp were clustered into 18,542 OTUs after the removal of singletons and following random subsampling of each sample to 3,100 sequences. There were 21 phyla and 120 genera detected among all intestinal samples. Overall, the ten most abundant OTUs accounted for 27.9% of sequences (data not shown). Independent of shedding status or intestinal section sampled, *Firmicutes* and *Bacteoidetes* comprised greater than 70% of all sequences (Table A in S1 File). There was considerably more variation among genera as only *Turcibacter* and *Prevotella* had an average relative abundance of greater than 4% among all samples (Table B in S1 File). The bacterial diversity in the bovine GI tract was highly variable and was strongly dependent on the GI site that the sample was derived from (Fig 1). There were no OTUs that were shared among all samples, although four OTUs were shared by 90% of the samples (Table C in S1 File). *Turcibacter*, *SMB83*, and *Clostridium* were the only genera that were detected in greater than 90% of all samples. Within the upper GI tract samples, only the genus *Mogibacterium* was found in each sample, while in the lower GI tract, *Turcibacter*, *Clostridium*, and *Ruminococcus* were common to all samples.

### Effect of *E*. *coli* O157:H7 shedding status on the bovine GI tract microbiota

**Fig 2:**
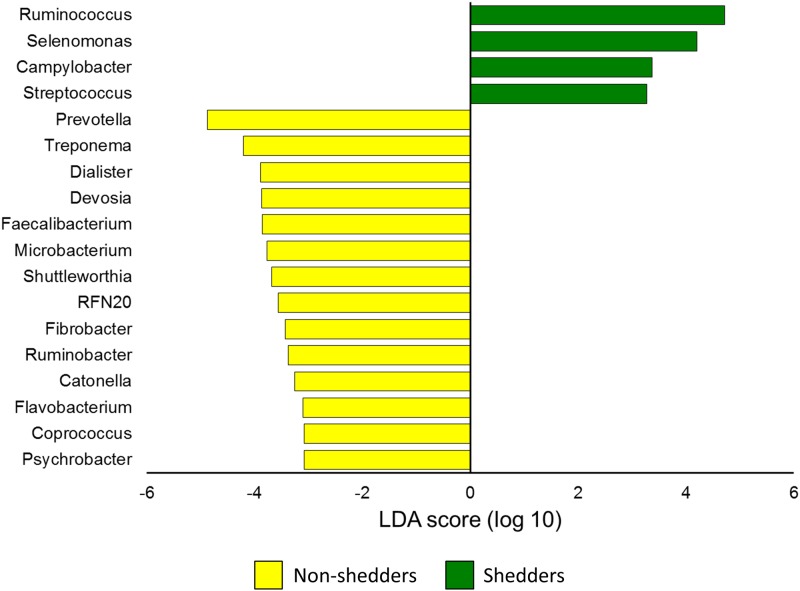
Differentially abundant genera associated with *E*. *coli* O157:H7 super-shedders as assessed using linear discriminant analysis effect size (LEfSe). Yellow bars indicate genera enriched in the non-shedder samples (n = 39) and green bars represent genera enriched in the shedder samples (n = 41). Only genera with a LDA score [log_10_] > 3.0 are displayed.

Differentially abundant phyla and genera between super-shedders and non-shedders were identified using LEfSe (LDA score [log_10_] > 3.0; P < 0.05) for pooled data of gastrointestinal samples. The non-shedders were found to have higher relative abundance of *Bacteroidetes*, *Fibrobacteres*, and *Cyanobacteria* phyla (LDA score [log_10_] > 3.5). At the genus level, *Prevotella* and *Treponema* were notably more abundant in non-shedders, while *Ruminococcocus*, *Selenomonas*, *Campylobacter*, and *Streptococcus* genera were enriched in super-shedders (Fig 2). Of these last four genera, only *Ruminococcus* had an overall relative abundance of greater than 0.05% (Table B in S1 File). When the GI section that the sample was derived from was included in the analysis, no difference was noted among the five and 20 most relatively abundant phyla and genera, respectively. This most likely reflects the high degree of variability, particularly at the genus-level among individual samples (Tables A and B in S1 File).

There were 12 OTUs that were differentially abundant between super-shedders and non-shedders in the lower GI samples (Table D in S1 File). Eight of these OTUs were more abundant in the non-shedder cohort and four in the super-shedder group. Similarly, in the upper GI samples, 12 OTUs were also differentially abundant between super-shedders and non-shedders with eight of these more abundant in the non-shedders (Table E in S1 File). Notably, two OTUs (514059 and 588315) were more abundant in super-shedders in both the upper and lower GI tract. These two OTUs were both classified as *Ruminococcus*, a genus which was also found to be more abundant in super-shedders using LEfSe. However, at the individual animal level, the enrichment of *Ruminococcus* within the super-shedding group appeared to be the result of one steer which had a relatively high proportion (> 26%) of this genus within the duodenum, proximal, mid, and distal jejunum, and the descending colon.

**Fig 3:**
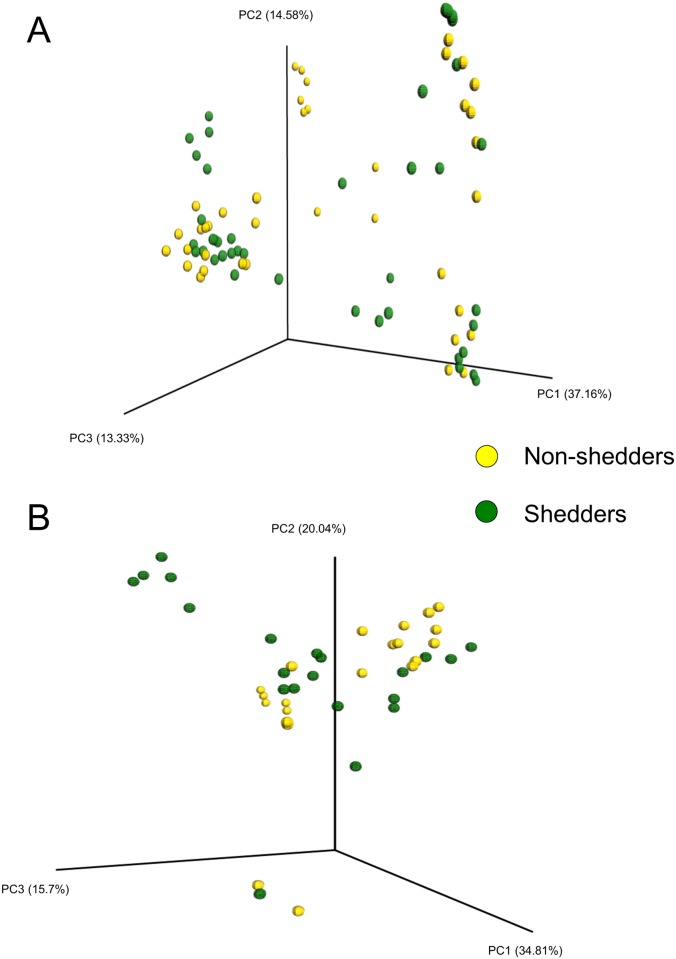
Principal coordinate analysis plots (PCoA) plots of the weighted UniFrac distances by *E*. *coli* O157:H7 shedding status. A) all samples and B) lower GI only. The percent variation explained by the principal coordinates is indicated on the axes.

Overall, in terms of bacterial diversity, none of the diversity or richness metrics differed significantly based on *E*. *coli* O157:H7 shedding status when lower and upper GI tract samples were combined (P > 0.05). However, there were significantly more OTUs and a higher Chao1 value in spiral colon samples from super-shedders compared with non-shedders (Fig 1A and 1B; P < 0.05). Although, not significant, the Shannon index and the phylogenetic diversity (PD whole tree) was also reduced in the non-shedders at this GI location (Fig 1C and 1D). A comparison of only the lower GI tract samples also revealed that super-shedders had significantly greater Chao1 values, OTU richness, and phylogenetic diversity than non-shedders (P < 0.05; data not shown). The bacterial community structure of samples from super-shedders and non-shedders was compared using weighted UniFrac distances. Samples did not cluster by shedding status when all samples were included in the analysis (Fig 3A; P > 0.05; R-value = 0.033).

When upper GI samples were removed from the analysis, there was a statistically significant difference between shedders and non-shedders (P = 0.014), but the associated R-value was relatively small (0.073) indicating that the two groups of samples were not well separated (Fig 3B). There were also 34 OTUs found in 90% of the lower GI samples from super-shedders (Table F in S1 File) while in the non-shedder lower GI samples, only seven OTUs were shared among 90% of the samples (Table G in S1 File). As the rectum and rectal tissue are most often believed to be the site of *E*. *coli* O157:H7 colonization, super-shedder and non-shedder samples taken from these two sites were compared using weighted UniFrac distances. In addition, due to the fact that spiral colon super-shedder and non-shedder samples differed based on alpha diversity metrics, this location was also isolated and analyzed using the same methods. However, the bacterial community structure at these three locations did not differ significantly based on *E*. *coli* O157:H7 shedding status (weighted UniFrac distances; data not shown; P > 0.05).

### Gut microbiota composition by gastrointestinal location

Whether a sample came from the upper (duodenum, proximal jejunum, mid-jejunum, distal jejunum) or lower (cecum, spiral colon, descending colon, rectal tissue, rectum) GI tract was the largest determinant of bacterial community composition. When samples were grouped by upper vs. lower GI tract, the upper GI tract samples had fewer OTUs, and lower Chao1, Shannon index, and PD whole tree values than the lower GI tract samples (Fig 1; P < 0.05). There were also 43 genera that were differentially abundant at one of the nine GI sections as assessed by LEfSe (Fig A in S2 File; LDA score [log_10_]> 3.0; P < 0.05). Among the most relatively abundant genera (> 1% of sequences overall), *Turicibacter* was enriched in the mid jejunum, *Prevotella* and *Lactobacillus* in the duodenum, SMB53 in the distal jejunum, *Clostridium* in the rectum, and CF231 and *Oscillospira* in the cecum. The number of OTUs shared by 100% of the samples from each GI section varied from only two in the distal jejunum to 45 OTUs in rectal samples (Table H in S1 File).

Not surprisingly, samples from the upper and lower GI tract formed two distinct clusters when analyzed using weighted UniFrac distances and PCoA (Fig B in S2 File; P < 0.001; R-value = 0.57). When the GI section that the sample was derived from was included in the analysis, the samples still clustered largely by GI tract location (upper and lower) rather than by individual GI section, although some duodenum samples appeared to cluster between the lower GI tract samples and the other upper GI tract samples (Fig C(A) in S2 File; P > 0.05). In the lower GI tract, samples clustered together mostly on the basis of host rather than on anatomical location within the GI tract (Fig C(B) in S2 File; P < 0.001; R-value = 0.67).

## Discussion

Changes in bacterial community structure along the GI tract has been well documented in humans [39, 40, 41] and more recently in other animals such as cats [42], horses [43] and dairy cows [44] using fingerprinting methods or 16S rRNA gene clone libraries. These studies highlight a clear longitudinal effect on dominant bacterial populations along the digestive tract of mammals. de Oliveira *et al*., [45] reported for the first time the use of pyrosequencing to characterize these changes along the entire gastrointestinal tract of one steer, from the rumen to the feces. The GI tract microbiota of dairy cattle has also recently been described based on high-throughput sequencing of the 16S rRNA gene [46]. In the present study, we used a similar approach to characterize changes in bacterial diversity across eight adjacent intestinal sections of ten feedlot steers, from the duodenum to the rectal-anal junction.

We observed that samples clustered largely based on whether they were derived from the upper or lower GI tract (Fig Ca in S2 File). Bacterial diversity and richness were significantly higher in the lower GI tract than in the upper GI tract where the *Firmicutes* phylum was largely predominant (> 85% of sequences), with the notable exception of the duodenum (Fig 1; Table A in S1 File). With the exception of samples taken from the spiral colon, a number of genera were enriched within the individual GI sections, including some of the most relatively abundant genera (Table B in S1 File). For example, *Prevotella* was enriched in the duodenum and *Turicibacter* in the mid jejunum. *Prevotella* spp., which are acetate producers, are also typically among the most abundant bacteria in the rumen [47, 48] and as the duodenum is the first site of pH neutrality in the GI tract after the abomasum, it seems likely that rumen digesta serves as a source of this genus. Although *Turicibacter* has been described as being relatively abundant in the cecum [49], colon [50], and feces [51] of feedlot cattle, it has also been reported to be enriched in the ileum and jejunum of dairy cattle [46].

The environmental conditions in the GI tract such as digesta pH (reduced in the upper GI tract), passage time (faster in the upper GI tract) or nutrient availability and composition, are all factors that may account for differences in microbiota along the GI tract. Furthermore, our results suggest that the bacterial microbiota is relatively stable along the bovine lower GI tract with more variability in the upper intestine. This finding is in agreement with previous observations in both cattle [45], humans [41], and pigs [52].

In a previous study, we determined that super-shedders and non-shedder feedlot steers had a unique fecal microbiota [22]. This suggested that there may be a relationship between changes in the bovine GI tract microbiota and colonization by *E*. *coli* O157:H7. This bacterium is preferentially detected in the lower GI tract of cattle, and more precisely within the RAJ [14, 15]. Interestingly, the only significant difference in the microbiota of super-shedders and non-shedders along the length of the GI tract was observed in the spiral colon where super-shedders had greater OTU richness (Fig 1). However, the bacterial community structure was not different between the two groups of cattle at this GI location. Although *Ruminococcus* appeared to be enriched in the GI microbiota of super-shedders based on LEfSe (Fig 2), analysis of individual steers revealed that this difference was largely the result of one steer having a high proportion of *Ruminococcus* at several anatomical locations within the GI tract.

*Prevotella* and *Treponema* genera were both markedly enriched in the GI tract of the non-shedder cattle. Within this cohort, *Prevotella* was relatively most abundant in the duodenum, cecum, rectum, and rectal tissue (Table B in S1 File). In addition to being relatively abundant in the rumen of cattle, *Prevotella* spp. are also significant members of the lower GI tract and fecal microbiota of dairy and feedlot cattle [26, 51, 53]. Certain *Treponema* spp. have been implicated in diseases in cattle such as bovine digital dermatitis (BDD) and it has recently been suggested that the GI tract serves as a reservoir for BDD-associated *Treponema* spp. [54]. However, this genus is also ubiquitous and relatively abundant within the rumen and lower GI tract of healthy cattle as evidenced from previous microbiome studies [22, 26, 46, 53].

Recent work aimed at describing and understanding the human gut microbiota has demonstrated the importance of bacterial diversity in the resilience of the gut microbiota and its implication in the prevention of numerous diseases [55]. In particular, it is now generally believed that a more diverse microbiota is more stable and less susceptible to invasion by enteropathogens than one with lower diversity and richness [56, 57]. This is known as the colonization resistance mechanism. Indeed, colonization by pathogens responsible for intestinal inflammation is often associated with dysbiosis of the microbiota in humans. In most cases, bacterial richness is reduced in infected vs. healthy individuals. For example, this was observed in patients suffering from diarrhea caused by *Clostridium difficile* [58, 59], in mice infected with *Salmonella enterica* [60] or *Campylobacter jejuni* [61], in TLR5-KO mice infected with adherent-invasive *E*. *coli* [62], and in pigs infected with enterotoxigenic *Escherichia coli* strain F4 [63].

The loss of colonization resistance leading to the potential overgrowth of enteropathogens and to gut inflammation might be due to several possible mechanisms mediated by complex ecological interactions [57]. One of the proposed mechanisms for this observation suggests that virulence factors enable the pathogen to elicit help from the host inflammatory response to gain a competitive growth advantage over resident microbiota [64]. We observed an increase in microbial diversity and richness in the spiral colon and overall in the lower GI tract of super-shedders (Fig 1), an observation that may reflect the uncertainty of the role of *E*. *coli* O157:H7 in intestinal inflammation in cattle [65]. The only similar report concerns helminth colonization of the human gut [66]. Perhaps, because intestinal inflammation in cattle is not as severe as in humans, the bovine immune response is different and as a result does not induce a similar perturbation of the microbiota. Further studies should attempt to define host immune responses in the large intestine of super-shedders, particularly given the recent finding that the expression of genes associated with immunity within the rectal tissue of super-shedders is suppressed [67]. The possibility also exists that differences in the bacterial microbiome within the spiral colon of super-shedders and non-shedders may be a reflection of digesta flow differences from the small intestine and cecum. No ileum samples were available for analysis in this study; however the spiral colon samples of super-shedders appeared to group more closely with their respective cecum samples than in non-shedders. In addition, both the PCoA and the alpha diversity analysis by section of the GI tract indicated more variability among the spiral colon samples in the non-shedders than in the super-shedders.

In conclusion, this study provides new insight into the total gut microbiota of cattle shedding *E*. *coli* O157:H7. Although the overall bacterial community structure was not altered by *E*. *coli* O157:H7 shedding status, several major bacterial genera, such as *Prevotella* and *Treponema*, as well as the phylum *Bacteroidetes*, were differentially abundant in the two groups of cattle. In addition, spiral colon samples from super-shedders had greater bacterial richness than non-shedders. Despite the fact that super-shedding status is usually defined by *E*. *coli* O157:H7 shedding concentrations in the rectum, the microbiota of this GI section and that of the rectal tissue samples was not different between super-shedders and non-shedders.

## Supporting information

S1 File Tables supporting information. Table A in S1 File. Comparisons of the five most relatively abundant phyla by gastrointestinal section and *E*. *coli* O157:H7 shedding status.
Table B in S1 File. Comparisons of the 20 most relatively abundant genera by gastrointestinal section and *E*. *coli* O157:H7 shedding status.
Table C in S1 File. OTUs found in 90% of all samples (n = 80).
Table D in S1 File. Differentially abundant OTUs between super-shedders (n = 21) and non-shedders (n = 21) in lower GI samples.
Table E in S1 File. Differentially abundant OTUs between super-shedders (n = 20) and non-shedders (n = 18) in upper GI samples.
Table F in S1 File. OTUs found in 90% of all lower GI samples from super-shedding cattle (n = 21).
Table G in S1 File. OTUs found in 90% of all lower GI samples from non-shedding cattle (n = 21).
Table H in S1 File. OTUs found in 100% of the samples taken from each GI section. n refers to the number of samples from each GI section.
(PDF)

S2 File Figures supporting information. Fig A in S2 File. Differentially abundant genera in each gastrointestinal section as assessed using LEfSE.
Fig B in S2 File. PCoA plot of the weighted UniFrac distances for lower and upper gastrointestinal samples.
Fig C (A) in S2 File. PCoA plots of the weighted UniFrac distances for each gastrointestinal section.
Fig C (B) in S2 File. PCoA plots of the weighted UniFrac distances for each animal for lower GI samples only
(PDF)
