# Correction: Changes in bacterial community composition of *Escherichia coli* O157:H7 super-shedder cattle occur in the lower intestine

**DOI:** 10.1371/journal.pone.0172387

**Published:** 2017-02-13

**Authors:** Rahat Zaheer, Eric Dugat-Bony, Devin B. Holman, Elodie Cousteix, Yong Xu, Krysty Munns, Lorna J. Selinger, Rutn Barbieri, Trevor Alexander, Tim A. McAllister, L. Brent Selinger

The third author’s name is spelled incorrectly. The correct name is: Devin B. Holman. The correct citation is: Zaheer R, Dugat-Bony E, Holman DB, Cousteix E, Xu Y, Munns K, et al. (2017) Changes in bacterial community composition of *Escherichia coli* O157:H7 super-shedder cattle occur in the lower intestine. PLoS ONE 12(1): e0170050. doi:10.1371/journal.pone.0170050
