# Supplementary material for: Changes in bacterial community composition of Escherichia coli O157:H7 super-shedder cattle occur in the lower intestine
Source: PLoS One. 2017 Jan 31;12(1):e0170050. doi: 10.1371/journal.pone.0170050 (PMC5283656; doi:10.1371/journal.pone.0170050)
Supplement: S1 File — Table A in S1 File. Comparisons of the five most relatively abundant phyla by gastrointestinal section and E. coli O157:H7 shedding status. Table B in S1 File. Comparisons of the 20 most relatively abundant genera by gastrointestinal section and E. coli O157:H7 shedding status. Table C in S1 File. OTUs found in 90% of all samples (n = 80). Table D in S1 File. Differentially abundant OTUs between super-shedders (n = 21) and non-shedders (n = 21) in lower GI samples. Table E in S1 File. Differentially abundant OTUs between super-shedders (n = 20) and non-shedders (n = 18) in upper GI samples. Table F in S1 File. OTUs found in 90% of all lower GI samples from super-shedding cattle (n = 21). Table G in S1 File. OTUs found in 90% of all lower GI samples from non-shedding cattle (n = 21). Table H in S1 File. OTUs found in 100% of the samples taken from each GI section. n refers to the number of samples from each GI section. (PDF) [file pone.0170050.s001.pdf]

Supporting Tables

Table A. Comparisons of the five most relatively abundant phyla by gastrointestinal section and *E. coli* O157:H7 shedding status. N=5 for all groups except: n= 3 for distal jejunum NS, cecum NS, descending colon SS, rectal tissue NS, and n=4 for rectal tissue SS, Rectum SS. Values represent mean relative abundance ± STD. NS=non-shedder, SS=super-shedder. Different uppercase letters across rows indicate significant differences among gastrointestinal sections and *E. coli* O157:H7 shedding status (P < 0.05). ND= not detected.

	**Duodenum**		**Proximal jejunum**		**Mid jejunum**		**Distal jejunum**		**Cecum**		**Spiral colon**		**Descending colon**		**Rectal tissue**		**Rectum**	
	**NS**	**SS**	**NS**	**SS**	**NS**	**SS**	**NS**	**SS**	**NS**	**SS**	**NS**	**SS**	**NS**	**SS**	**NS**	**SS**	**NS**	**SS**
Firmicutes	55.0±24.1	71.5±31.3	88.6±18.1	94.5±3.0	82.0±31.3	97.7±2.1A	96.0±3.9	95.9±3.3	49.8±18.5	62.7±7.8	60.7±26.0	53.5±11.6	53.6±12.5	60.4±25.4	44.2±15.7	50.3±11.2	53.2±17.1	63.5±15.6
	BCD	ABCD	ABCD	ABC	ABCD		ABCD	AB	ABCD	ABCD	ABCD	CD	CD	ABCD	D	CD	CD	ABCD
Bacteroidetes	31.2±18.3	5.8±6.2	5.1±9.7	0.09±0.14	3.5±6.1	0.04±0.06	0.47±0.58	0.17±0.25	36.4±17.1	25.7±9.7	24.0±19.4	30.8±14.2	29.3±10.7	28.2±24.9	35.7±20.9	32.1±13.9	32.6±16.2	24.3±15.8
	A	AB	AB	B	AB	B	AB	B	A	AB	AB	A	AB	AB	A	A	A	AB
Spirochaetes	1.75±3.58	0.46±0.72	0.11±0.15	0.01±0.01	0.10±0.13	ND	0.06±0.06	0.03±0.04	4.0±6.8	4.6±5.5	7.4±10.7	9.0±11.1	9.9±9.8	1.1±1.8	8.9±9.6	9.0±11.1	6.8±6.9	3.7±5.71
Proteobacteria	6.2±6.4	16.0±30.1	1.4±2.8	0.85±1.03	1.4±1.7	0.66±0.98	0.46±0.16	0.92±1.53	4.0±6.6	1.7±1.3	4.3±5.5	2.1±2.1	3.1±4.1	1.6±2.3	7.9±5.7	3.4±1.8	2.9±4.5	0.94±1.26
Tenericutes	0.19±0.13	1.2±2.1	1.5±3.1	0.06±0.06	10.3±22.5	0.11±0.12	0.24±0.25	0.04±0.05	0.69±0.30	0.19±0.13	1.0±1.7	0.30±0.27	0.50±0.32	0.71±0.69	0.17±0.09	0.26±0.12	0.41±0.34	0.27±0.27

Table B. Comparisons of the 20 most relatively abundant genera by gastrointestinal section and *E. coli* O157:H7 shedding status. N=5 for all groups except: n= 3 for distal jejunum NS, cecum NS, descending colon SS, rectal tissue NS, and n=4 for rectal tissue SS, Rectum SS. Values represent mean relative abundance ± STD. NS=non-shedder, SS=super-shedder. Different uppercase letters across rows indicate significant differences among gastrointestinal sections and *E. coli* O157:H7 shedding status ($P < 0.05$). ND=not detected.

	Duodenum		Proximal jejunum		Mid jejunum		Distal jejunum		Cecum		Spiral colon		Descending colon		Rectal tissue		Rectum	
	NS	SS	NS	SS	NS	SS	NS	SS	NS	SS	NS	SS	NS	SS	NS	SS	NS	SS
Turicibacter	0.43±0.19	0.92±0.42	11.1±14.8	6.8±10.2	9.2±11.3	14.2±9.5	10.3±6.9	10.3±11.8	0.43±0.19	0.92±0.42	1.7±1.3	0.88±0.54	0.79±0.54	0.72±0.70	0.63±0.38	0.63±0.39	0.77±0.45	0.64±0.46
Prevotella	14.1±23.8	2.97±2.66	1.5±2.6	0.02±0.03	1.3±1.8	0.04±0.06	0.03±0.06	0.05±0.08	14.1±23.8	3.0±2.7	9.2±13.2	3.7±4.3	7.3±12.9	3.6±4.8	18.8±25.2	3.4±1.6	10.4±18.2	2.4±3.6
	A	AB	B	B	B	B	AB	B	AB	AB	AB	AB	AB	AB	AB	AB	AB	AB
Treponema	4.0±6.8	4.64±5.51	0.11±0.15	0.01±0.01	0.10±0.13	ND	0.06±0.06	0.03±0.04	4.0±6.8	4.6±5.5	7.4±10.7	9.0±11.1	9.8±9.8	1.1±1.8	8.9±9.6	9.0±11.1	6.8±6.9	3.6±5.7
Ruminococcus	0.55±0.06	1.49±1.77	0.35±0.54	9.0±16.1	0.12±0.17	5.9±12.5	0.32±0.56	17.8±36.0	0.55±0.06	1.5±1.8	0.75±0.75	0.97±1.22	0.65±0.48	17.4±29.5	1.9±2.4	0.58±0.51	0.61±0.40	0.69±0.36
5-7N15	3.7±3.5	5.7±4.0	0.77±1.56	ND	0.46±0.97	ND	0.08±0.13	0.01±0.02	3.7±3.5	5.7±4.0	3.2±4.4	7.0±3.9	5.5±3.4	6.2±6.5	4.8±3.7	8.3±4.0	5.0±3.3	5.6±3.2
	C	C	BC		BC		BC	C	ABC	ABC	ABC	AB	C	C	ABC	AB	ABC	ABC
CF231	3.5±3.4	4.90±2.99	0.35±0.74	ND	0.16±0.31	ND	0.04±0.04	0.01±0.01	3.5±3.4	4.9±3.0	3.6±5.6	5.5±2.8	3.8±3.5	2.6±2.2	1.9±1.3	3.8±2.0	5.0±4.8	3.7±0.9
SMB53	0.12±0.10	0.35±0.23	5.4±4.9	4.8±5.1	4.7±4.6	7.2±5.6	6.0±4.4	4.7±3.6	0.12±0.10	0.35±0.23	0.85±0.93	0.29±0.22	0.26±0.17	0.20±0.05	0.16±0.03	0.11±0.06	0.17±0.07	0.18±0.08
	B	B	AB	AB	AB	A	AB	AB	AB	B	AB	B	B	AB	AB	B	B	B
Clostridium	2.9±2.3	3.71±0.89	0.35±0.55	0.13±0.11	0.21±0.28	0.15±0.10	0.53±0.05	0.15±0.17	2.9±2.3	3.7±0.9	1.5±1.9	2.8±1.1	3.1±2.0	1.6±1.6	1.8±1.2	2.6±0.8	3.2±1.8	4.1±1.4
	D	BCD	CD	D	D	D	BCD	D	ABCD	D	ABCD	ABC	AB	ABCD	ABCD	ABCD	AB	A
Oscillospira	1.41±0.46	3.49±2.18	0.70±1.20	0.05±0.10	0.36±0.51	0.06±0.07	0.30±0.52	0.02±0.04	1.41±0.46	3.5±2.2	1.0±0.6	2.7±2.0	2.0±0.7	4.8±2.2	2.0±0.5	2.4±0.5	1.6±0.6	2.4±1.0
	B	AB	AB	B	B	B	AB	B	AB	AB	AB	AB	AB	A	AB	AB	AB	AB
Lactobacillus	ND	0.21±0.43	0.99±0.85	2.6±3.3	1.8±2.8	0.27±0.24	0.90±0.78	3.8±6.6	0.000.00	0.21±0.43	1.3±2.5	0.05±0.07	0.04±0.04	0.65±1.09	0.03±0.06	ND	0.03±0.06	0.02±0.02
Helicobacter	0.03±0.06	0.00±0.00	0.03±0.04	0.09±0.17	0.01±0.01	0.11±0.25	0.01±0.02	0.05±0.07	0.03±0.06	ND	0.08±0.11	ND	0.01±0.01	ND	ND	ND	ND	ND
Butyrivibrio	0.13±0.22	0.03±0.04	0.63±0.96	3.0±3.8	0.44±0.65	0.72±1.30	1.0±1.6	0.92±1.52	0.13±0.22	0.03±0.04	0.43±0.85	0.02±0.03	0.01±0.02	0.30±0.47	ND	0.02±0.05	0.01±0.01	ND
	AB	AB	AB	AB	AB	AB	AB	AB	AB	B	AB	B	AB	B		AB	B	
Succinivibrio	1.6±2.8	1.06±1.51	0.02±0.04	0.01±0.01	0.09±0.20	ND	ND	0.01±0.01	1.6±2.8	1.1±1.5	2.1±4.0	1.0±2.0	1.6±3.0	0.04±0.05	3.7±3.0	0.53±0.77	1.9±3.7	0.14±0.21
Bulleidia	0.10±0.12	0.12±0.18	1.2±1.6	2.1±3.1	1.2±1.3	0.57±0.71	1.20±0.89	0.83±1.22	0.10±0.12	0.12±0.18	0.66±1.19	0.06±0.07	0.05±0.05	0.05±0.05	0.11±0.13	0.10±0.14	0.01±0.03	0.18±0.21
Mogibacterium	0.13±0.06	0.08±0.12	1.4±1.5	1.4±1.1	1.6±2.5	0.84±1.36	0.98±1.05	0.83±0.78	0.13±0.06	0.08±0.12	0.59±0.89	0.05±0.06	0.06±0.05	0.66±1.08	0.09±0.15	0.02±0.02	0.11±0.04	0.06±0.07
Dialister	0.69±1.14	0.02±0.03	0.14±0.19	0.06±0.06	0.22±0.22	ND	0.03±0.03	0.03±0.04	0.69±1.14	0.02±0.03	0.34±0.45	ND	0.21±0.44	ND	0.46±0.80	0.01±0.02	0.21±0.46	0.34±0.68
	A	B	B	B	B		B	B	B	B	B		B		B	B	B	B
Sutterella	0.52±0.84	0.45±0.58	0.07±0.16	ND	0.03±0.06	ND	0.01±0.02	ND	0.52±0.84	0.45±0.58	0.89±1.55	0.85±1.31	0.74±1.16	0.68±1.09	1.41.2	2.1±2.2	0.56±0.85	0.30±0.53
Phascolarctobac	0.51±0.47	1.23±1.23	0.21±0.46	ND	0.06±0.14	ND	ND	ND	0.51±0.47	1.2±1.2	0.11±0.14	1.2±0.7	0.31±0.17	0.72±0.89	0.98±1.28	1.4±1.3	0.52±0.35	1.1±0.7
	B	B	AB		AB				AB	AB	AB	AB	AB	AB	AB	A	AB	AB
Anaerovibrio	0.11±0.10	0.94±1.36	ND	ND	0.02±0.04	ND	ND	ND	0.11±0.10	0.94±1.36	0.08±0.13	1.8±2.3	0.23±0.14	0.41±0.60	1.3±1.3	2.0±3.6	0.12±0.14	0.37±0.62
Faecalibacterium	1.1±1.9	ND	0.06±0.14	ND	0.01±0.01	ND	ND	ND	1.1±1.9	ND	1.5±3.2	ND	1.5±3.2	ND	0.90±1.56	ND	1.1±2.4	0.01±0.02

Table C. OTUs found in 90% of all samples (n=80).

OTU ID	Greengenes taxonomy
347575	['k__Bacteria', 'p__Firmicutes', 'c__Clostridia', 'o__Clostridiales', 'f__Peptostreptococcaceae
813277	['k__Bacteria', 'p__Firmicutes', 'c__Clostridia', 'o__Clostridiales', 'f__Ruminococcaceae
341322	['k__Bacteria', 'p__Firmicutes', 'c__Bacilli', 'o__Turicibacterales', 'f__Turicibacteraceae', 'g__Turicibacter
308502	['k__Bacteria', 'p__Firmicutes', 'c__Clostridia', 'o__Clostridiales', 'f__Peptostreptococcaceae

Table D. Differentially abundant OTUs between super-shedders (n=21) and non-shedders (n=21) in lower GI samples. Average abundance values for each OTU are reported. FDR (false discovery rate) < 0.05.

OTU ID	FDR	Non-shedders	Super-shedders	Greengenes taxonomy
514059	0	0.048	60.38	k__Bacteria; p__Firmicutes; c__Clostridia; o__Clostridiales; f__Ruminococcaceae; g__Ruminococcus
178387	4.79×10^{-10}	46.90	0.95	k__Bacteria; p__Bacteroidetes; c__Bacteroidia; o__Bacteroidales; f__Prevotellaceae; g__Prevotella; s__copri
813277	4.79×10^{-10}	93.62	18.0	k__Bacteria; p__Firmicutes; c__Clostridia; o__Clostridiales; f__Ruminococcaceae
529442	6.50×10^{-10}	1.29	47.14	k__Bacteria; p__Bacteroidetes; c__Bacteroidia; o__Bacteroidales; f__Rikenellaceae
20534	8.43×10^{-9}	142.76	47.90	k__Bacteria; p__Bacteroidetes; c__Bacteroidia; o__Bacteroidales; f__[Paraprevotellaceae]; g__[Prevotella]
288265	3.81×10^{-8}	46.33	2.86	k__Bacteria; p__Bacteroidetes; c__Bacteroidia; o__Bacteroidales; f__Prevotellaceae
522371	0.0002	3.19	33.62	k__Bacteria; p__Bacteroidetes; c__Bacteroidia; o__Bacteroidales; f__Rikenellaceae_
OTU1	0.0033	44.19	9.90	k__Bacteria; p__Firmicutes; c__Clostridia; o__Clostridiales; f__Ruminococcaceae
517804	0.0070	48.62	13.10	k__Bacteria; p__Proteobacteria; c__Gammaproteobacteria; o__Aeromonadales; f__Succinivibrionaceae; g__Succinivibrio
163648	0.0106	180.76	104.52	k__Bacteria; p__Spirochaetes; c__Spirochaetes; o__Spirochaetales; f__Spirochaetaceae; g__Treponema_
588315	0.0143	0.048	15.19	k__Bacteria; p__Firmicutes; c__Clostridia; o__Clostridiales; f__Ruminococcaceae; g__Ruminococcus
801210	0.0286	15.190	0.238	k__Bacteria; p__Proteobacteria; c__Betaproteobacteria; o__Burkholderiales; f__Alcaligenaceae; g__Sutterella

Table E. Differentially abundant OTUs between super-shedders (n=20) and non-shedders (n=18) in upper GI samples. FDR (false discovery rate) < 0.05.

OTU ID	FDR	Non-shedders	Super-shedders	Greengenes taxonomy
514059	0	0.056	201.85	k__Bacteria; p__Firmicutes; c__Clostridia; o__Clostridiales; f__Ruminococcaceae; g__Ruminococcus
10980	0	1.89	104.65	k__Bacteria; p__Proteobacteria; c__Epsilonproteobacteria; o__Campylobacterales; f__Helicobacteraceae; g__Helicobacter
4383090	6.86×10^{-13}	65.39	3.0	k__Bacteria; p__Bacteroidetes; c__Bacteroidia; o__Bacteroidales; f__Prevotellaceae; g__Prevotella
588315	4.01×10^{-11}	0.056	44.45	k__Bacteria; p__Firmicutes; c__Clostridia; o__Clostridiales; f__Ruminococcaceae; g__Ruminococcus
OTU125	6.88×10^{-11}	71.44	7.75	k__Bacteria; p__Tenericutes; c__Mollicutes; o__Mycoplasmatales; f__Mycoplasmataceae
570232	3.05×10^{-6}	28.28	0.1	k__Bacteria; p__Tenericutes; c__Mollicutes; o__Mycoplasmatales; f__Mycoplasmataceae; g__Mycoplasma
OTU16	0.0010	31.228	3.3	k__Bacteria; p__Firmicutes; c__Clostridia; o__Clostridiales; f__Veillonellaceae; g__Dialister
815378	0.0028	25.948	2.1	k__Bacteria; p__Proteobacteria; c__Gammaproteobacteria; o__Aeromonadales; f__Succinivibrionaceae
816696	0.0093	21.39	1.3	k__Bacteria; p__Proteobacteria; c__Gammaproteobacteria; o__Aeromonadales; f__Succinivibrionaceae
OTU145	0.017	0.119	15.5	k__Bacteria; p__TM7; c__TM7-3; o__CW040; f__F16
303445	0.018	16.28	0.3	k__Bacteria; p__Bacteroidetes; c__Bacteroidia; o__Bacteroidales; f__p-2534-18B5
545922	0.048	15.83	0.55	k__Bacteria; p__Bacteroidetes; c__Bacteroidia; o__Bacteroidales; f__Prevotellaceae; g__Prevotella

Table F. OTUs found in 90% of all lower GI samples from super-shedding cattle (n=21).

OTU ID	Greengenes taxonomy
297143	Bacteria', 'p__Firmicutes', 'c__Clostridia', 'o__Clostridiales
4446320	Bacteria', 'p__Firmicutes', 'c__Clostridia', 'o__Clostridiales', 'f__Clostridiaceae
330414	Bacteria', 'p__Firmicutes', 'c__Clostridia', 'o__Clostridiales', 'f__Lachnospiraceae
526676	Bacteria', 'p__Bacteroidetes', 'c__Bacteroidia', 'o__Bacteroidales', 'f__S24-7
304568	Bacteria', 'p__Firmicutes', 'c__Clostridia', 'o__Clostridiales', 'f__Lachnospiraceae
297769	Bacteria', 'p__Bacteroidetes', 'c__Bacteroidia', 'o__Bacteroidales', 'f__Bacteroidaceae', 'g__5-7N15
355912	Bacteria', 'p__Firmicutes', 'c__Clostridia', 'o__Clostridiales', 'f__Clostridiaceae', 'g__Clostridium
300611	Bacteria', 'p__Firmicutes', 'c__Clostridia', 'o__Clostridiales', 'f__Ruminococcaceae
573068	Bacteria', 'p__Firmicutes', 'c__Clostridia', 'o__Clostridiales', 'f__Ruminococcaceae
310725	Bacteria', 'p__Firmicutes', 'c__Clostridia', 'o__Clostridiales
347575	Bacteria', 'p__Firmicutes', 'c__Clostridia', 'o__Clostridiales', 'f__Peptostreptococcaceae
319662	Bacteria', 'p__Bacteroidetes', 'c__Bacteroidia', 'o__Bacteroidales', 'f__[Paraprevotellaceae]', 'g__CF231
186092	Bacteria', 'p__Firmicutes', 'c__Clostridia', 'o__Clostridiales', 'f__Clostridiaceae
584083	Bacteria', 'p__Firmicutes', 'c__Clostridia', 'o__Clostridiales
813277	Bacteria', 'p__Firmicutes', 'c__Clostridia', 'o__Clostridiales', 'f__Ruminococcaceae
302013	Bacteria', 'p__Bacteroidetes', 'c__Bacteroidia', 'o__Bacteroidales', 'f__[Paraprevotellaceae]
341322	Bacteria', 'p__Firmicutes', 'c__Bacilli', 'o__Turicibacterales', 'f__Turicibacteraceae', 'g__Turicibacter
334216	Bacteria', 'p__Firmicutes', 'c__Clostridia', 'o__Clostridiales', 'f__Ruminococcaceae
314304	Bacteria', 'p__Firmicutes', 'c__Clostridia', 'o__Clostridiales', 'f__Lachnospiraceae']
304482	Bacteria', 'p__Bacteroidetes', 'c__Bacteroidia', 'o__Bacteroidales', 'f__[Paraprevotellaceae]', 'g__CF231
287875	Bacteria', 'p__Firmicutes', 'c__Clostridia', 'o__Clostridiales', 'f__Ruminococcaceae
308502	Bacteria', 'p__Firmicutes', 'c__Clostridia', 'o__Clostridiales', 'f__Peptostreptococcaceae
518013	Bacteria', 'p__Bacteroidetes', 'c__Bacteroidia', 'o__Bacteroidales', 'f__Bacteroidaceae', 'g__5-7N15
319354	Bacteria', 'p__Bacteroidetes', 'c__Bacteroidia', 'o__Bacteroidales', 'f__Bacteroidaceae', 'g__5-7N15
299860	Bacteria', 'p__Firmicutes', 'c__Clostridia', 'o__Clostridiales', 'f__Ruminococcaceae
319981	Bacteria', 'p__Firmicutes', 'c__Clostridia', 'o__Clostridiales
317802	Bacteria', 'p__Bacteroidetes', 'c__Bacteroidia', 'o__Bacteroidales', 'f__Bacteroidaceae', 'g__5-7N15
323794	Bacteria', 'p__Firmicutes', 'c__Clostridia', 'o__Clostridiales', 'f__Lachnospiraceae
528782	Bacteria', 'p__Firmicutes', 'c__Clostridia', 'o__Clostridiales', 'f__Ruminococcaceae
4415144	Bacteria', 'p__Bacteroidetes', 'c__Bacteroidia', 'o__Bacteroidales', 'f__Bacteroidaceae', 'g__5-7N15
293937	Bacteria', 'p__Firmicutes', 'c__Clostridia', 'o__Clostridiales', 'f__Clostridiaceae', 'g__Clostridium
289992	Bacteria', 'p__Firmicutes', 'c__Clostridia', 'o__Clostridiales
OTU163	Bacteria', 'p__Firmicutes', 'c__Clostridia', 'o__Clostridiales', 'f__Ruminococcaceae
OTU48	Bacteria', 'p__Firmicutes', 'c__Clostridia', 'o__Clostridiales', 'f__Ruminococcaceae

Table G. OTUs found in 90% of all lower GI samples from non-shedding cattle (n=21).

OTU ID	Greengenes taxonomy
297143	Bacteria', 'p__Firmicutes', 'c__Clostridia', 'o__Clostridiales
368490	Bacteria', 'p__Firmicutes', 'c__Bacilli', 'o__Turicibacterales', 'f__Turicibacteraceae', 'g__Turicibacter
4446320	Bacteria', 'p__Firmicutes', 'c__Clostridia', 'o__Clostridiales', 'f__Clostridiaceae
813277	Bacteria', 'p__Firmicutes', 'c__Clostridia', 'o__Clostridiales', 'f__Ruminococcaceae
341322	Bacteria', 'p__Firmicutes', 'c__Bacilli', 'o__Turicibacterales', 'f__Turicibacteraceae', 'g__Turicibacter
308502	Bacteria', 'p__Firmicutes', 'c__Clostridia', 'o__Clostridiales', 'f__Peptostreptococcaceae
590980	Bacteria', 'p__Firmicutes', 'c__Clostridia', 'o__Clostridiales', 'f__Ruminococcaceae', 'g__Oscillospira

Table H. OTUs found in 100% of the samples taken from each GI section. n refers to the number of samples from each GI section.

**Duodenum**			n=10
	646411	k__Bacteria', 'p__Firmicutes', 'c__Erysipelotrichi', 'o__Erysipelotrichales', 'f__Erysipelotrichaceae', 'g__Bulleidia	
	553813	k__Bacteria', 'p__Firmicutes', 'c__Clostridia', 'o__Clostridiales', 'f__Lachnospiraceae', 'g__Butyrivibrio	
	594419	k__Bacteria', 'p__Firmicutes', 'c__Clostridia', 'o__Clostridiales	
	813277	k__Bacteria', 'p__Firmicutes', 'c__Clostridia', 'o__Clostridiales', 'f__Ruminococcaceae', 'g__	
	806057	k__Bacteria', 'p__Firmicutes', 'c__Clostridia', 'o__Clostridiales	
New.ReferenceOTU1		k__Bacteria', 'p__Firmicutes', 'c__Clostridia', 'o__Clostridiales', 'f__Ruminococcaceae', 'g__	
**Proximal jejunum**			n=10
	368490	k__Bacteria', 'p__Firmicutes', 'c__Bacilli', 'o__Turicibacterales', 'f__Turicibacteraceae', 'g__Turicibacter	
	347575	k__Bacteria', 'p__Firmicutes', 'c__Clostridia', 'o__Clostridiales', 'f__Peptostreptococcaceae', 'g__	
	813277	k__Bacteria', 'p__Firmicutes', 'c__Clostridia', 'o__Clostridiales', 'f__Ruminococcaceae', 'g__	
	341322	k__Bacteria', 'p__Firmicutes', 'c__Bacilli', 'o__Turicibacterales', 'f__Turicibacteraceae', 'g__Turicibacter	
	173883	k__Bacteria', 'p__Firmicutes', 'c__Clostridia', 'o__Clostridiales', 'f__Clostridiaceae', 'g__SMB53	
	3894019	k__Bacteria', 'p__Firmicutes', 'c__Clostridia', 'o__Clostridiales', 'f__Clostridiaceae', 'g__SMB53	
	806057	k__Bacteria', 'p__Firmicutes', 'c__Clostridia', 'o__Clostridiales	
New.ReferenceOTU1		k__Bacteria', 'p__Firmicutes', 'c__Clostridia', 'o__Clostridiales', 'f__Ruminococcaceae', 'g__	
**Mid jejunum**			n=10
	297143	k__Bacteria', 'p__Firmicutes', 'c__Clostridia', 'o__Clostridiales	
	368490	k__Bacteria', 'p__Firmicutes', 'c__Bacilli', 'o__Turicibacterales', 'f__Turicibacteraceae', 'g__Turicibacter	
	4446320	k__Bacteria', 'p__Firmicutes', 'c__Clostridia', 'o__Clostridiales', 'f__Clostridiaceae', 'g__	
	813277	k__Bacteria', 'p__Firmicutes', 'c__Clostridia', 'o__Clostridiales', 'f__Ruminococcaceae', 'g__	
	341322	k__Bacteria', 'p__Firmicutes', 'c__Bacilli', 'o__Turicibacterales', 'f__Turicibacteraceae', 'g__Turicibacter	
	184009	k__Bacteria', 'p__Firmicutes', 'c__Clostridia', 'o__Clostridiales', 'f__Clostridiaceae']	
	308502	k__Bacteria', 'p__Firmicutes', 'c__Clostridia', 'o__Clostridiales', 'f__Peptostreptococcaceae', 'g__	

	173883	k__Bacteria', 'p__Firmicutes', 'c__Clostridia', 'o__Clostridiales', 'f__Clostridiaceae', 'g__SMB53
	40639	k__Bacteria', 'p__Firmicutes', 'c__Clostridia', 'o__Clostridiales
	3894019	k__Bacteria', 'p__Firmicutes', 'c__Clostridia', 'o__Clostridiales', 'f__Clostridiaceae', 'g__SMB53
New.ReferenceOTU1		k__Bacteria', 'p__Firmicutes', 'c__Clostridia', 'o__Clostridiales', 'f__Ruminococcaceae', 'g__
**Distal jejnunum**		n=8
	368490	k__Bacteria', 'p__Firmicutes', 'c__Bacilli', 'o__Turicibacterales', 'f__Turicibacteraceae', 'g__Turicibacter
	813277	k__Bacteria', 'p__Firmicutes', 'c__Clostridia', 'o__Clostridiales', 'f__Ruminococcaceae', 'g__
**Cecum**		n=8
	297143	k__Bacteria', 'p__Firmicutes', 'c__Clostridia', 'o__Clostridiales']
	330414	k__Bacteria', 'p__Firmicutes', 'c__Clostridia', 'o__Clostridiales', 'f__Lachnospiraceae']
	526676	k__Bacteria', 'p__Bacteroidetes', 'c__Bacteroidia', 'o__Bacteroidales', 'f__S24-7', 'g__
	304568	k__Bacteria', 'p__Firmicutes', 'c__Clostridia', 'o__Clostridiales', 'f__Lachnospiraceae']
	297769	k__Bacteria', 'p__Bacteroidetes', 'c__Bacteroidia', 'o__Bacteroidales', 'f__Bacteroidaceae', 'g__5-7N15
	524111	k__Bacteria', 'p__Firmicutes', 'c__Clostridia', 'o__Clostridiales', 'f__Ruminococcaceae', 'g__
	355912	k__Bacteria', 'p__Firmicutes', 'c__Clostridia', 'o__Clostridiales', 'f__Clostridiaceae', 'g__Clostridium
	529083	k__Bacteria', 'p__Firmicutes', 'c__Clostridia', 'o__Clostridiales', 'f__Ruminococcaceae', 'g__
	584083	k__Bacteria', 'p__Firmicutes', 'c__Clostridia', 'o__Clostridiales
	813277	k__Bacteria', 'p__Firmicutes', 'c__Clostridia', 'o__Clostridiales', 'f__Ruminococcaceae', 'g__
	302013	k__Bacteria', 'p__Bacteroidetes', 'c__Bacteroidia', 'o__Bacteroidales', 'f__[Paraprevotellaceae]', 'g__CF231
	326382	k__Bacteria', 'p__Bacteroidetes', 'c__Bacteroidia', 'o__Bacteroidales', 'f__Bacteroidaceae', 'g__5-7N15
	334216	k__Bacteria', 'p__Firmicutes', 'c__Clostridia', 'o__Clostridiales', 'f__Ruminococcaceae
	525024	k__Bacteria', 'p__Firmicutes', 'c__Clostridia', 'o__Clostridiales', 'f__Clostridiaceae
	314304	k__Bacteria', 'p__Firmicutes', 'c__Clostridia', 'o__Clostridiales', 'f__Lachnospiraceae
	531310	k__Bacteria', 'p__Bacteroidetes', 'c__Bacteroidia', 'o__Bacteroidales', 'f__Bacteroidaceae', 'g__5-7N15
	304482	k__Bacteria', 'p__Bacteroidetes', 'c__Bacteroidia', 'o__Bacteroidales', 'f__[Paraprevotellaceae]', 'g__CF231
	308502	k__Bacteria', 'p__Firmicutes', 'c__Clostridia', 'o__Clostridiales', 'f__Peptostreptococcaceae
	518013	k__Bacteria', 'p__Bacteroidetes', 'c__Bacteroidia', 'o__Bacteroidales', 'f__Bacteroidaceae', 'g__5-7N15
	319354	k__Bacteria', 'p__Bacteroidetes', 'c__Bacteroidia', 'o__Bacteroidales', 'f__Bacteroidaceae', 'g__5-7N15
	323794	k__Bacteria', 'p__Firmicutes', 'c__Clostridia', 'o__Clostridiales', 'f__Lachnospiraceae', 'g__

	528782	k__Bacteria', 'p__Firmicutes', 'c__Clostridia', 'o__Clostridiales', 'f__Ruminococcaceae', 'g__	
	4415144	k__Bacteria', 'p__Bacteroidetes', 'c__Bacteroidia', 'o__Bacteroidales', 'f__Bacteroidaceae', 'g__5-7N15	
	293937	k__Bacteria', 'p__Firmicutes', 'c__Clostridia', 'o__Clostridiales', 'f__Clostridiaceae', 'g__Clostridium	
	289992	k__Bacteria', 'p__Firmicutes', 'c__Clostridia', 'o__Clostridiales	
	4311539	k__Bacteria', 'p__Bacteroidetes', 'c__Bacteroidia', 'o__Bacteroidales', 'f__S24-7', 'g__	
**Spiral colon**			n=10
	4446320	k__Bacteria', 'p__Firmicutes', 'c__Clostridia', 'o__Clostridiales', 'f__Clostridiaceae', 'g__	
	347575	k__Bacteria', 'p__Firmicutes', 'c__Clostridia', 'o__Clostridiales', 'f__Peptostreptococcaceae', 'g__	
	186092	k__Bacteria', 'p__Firmicutes', 'c__Clostridia', 'o__Clostridiales', 'f__Clostridiaceae', 'g__	
	341322	k__Bacteria', 'p__Firmicutes', 'c__Bacilli', 'o__Turicibacterales', 'f__Turicibacteraceae', 'g__Turicibacter	
	308502	k__Bacteria', 'p__Firmicutes', 'c__Clostridia', 'o__Clostridiales', 'f__Peptostreptococcaceae', 'g__	
**Descending colon**			n=8
	297143	k__Bacteria', 'p__Firmicutes', 'c__Clostridia', 'o__Clostridiales']	
	347575	k__Bacteria', 'p__Firmicutes', 'c__Clostridia', 'o__Clostridiales', 'f__Peptostreptococcaceae', 'g__	
	813277	k__Bacteria', 'p__Firmicutes', 'c__Clostridia', 'o__Clostridiales', 'f__Ruminococcaceae', 'g__	
	341322	k__Bacteria', 'p__Firmicutes', 'c__Bacilli', 'o__Turicibacterales', 'f__Turicibacteraceae', 'g__Turicibacter	
	308502	k__Bacteria', 'p__Firmicutes', 'c__Clostridia', 'o__Clostridiales', 'f__Peptostreptococcaceae', 'g__	
**Rectal tissue**			n=7
	305304	k__Bacteria', 'p__Proteobacteria', 'c__Betaproteobacteria', 'o__Burkholderiales', 'f__Alcaligenaceae', 'g__Sutterella	
	517804	k__Bacteria', 'p__Proteobacteria', 'c__Gammaproteobacteria', 'o__Aeromonadales', 'f__Succinivibrionaceae', 'g__Succinivibrio	
	4446320	k__Bacteria', 'p__Firmicutes', 'c__Clostridia', 'o__Clostridiales', 'f__Clostridiaceae', 'g__	
	330414	k__Bacteria', 'p__Firmicutes', 'c__Clostridia', 'o__Clostridiales', 'f__Lachnospiraceae']	
	526676	k__Bacteria', 'p__Bacteroidetes', 'c__Bacteroidia', 'o__Bacteroidales', 'f__S24-7', 'g__	
	304568	k__Bacteria', 'p__Firmicutes', 'c__Clostridia', 'o__Clostridiales', 'f__Lachnospiraceae']	
	297769	k__Bacteria', 'p__Bacteroidetes', 'c__Bacteroidia', 'o__Bacteroidales', 'f__Bacteroidaceae', 'g__5-7N15	
	355912	k__Bacteria', 'p__Firmicutes', 'c__Clostridia', 'o__Clostridiales', 'f__Clostridiaceae', 'g__Clostridium	
	300611	k__Bacteria', 'p__Firmicutes', 'c__Clostridia', 'o__Clostridiales', 'f__Ruminococcaceae', 'g__	

573068	k__Bacteria', 'p__Firmicutes', 'c__Clostridia', 'o__Clostridiales', 'f__Ruminococcaceae', 'g__
298163	k__Bacteria', 'p__Firmicutes', 'c__Clostridia', 'o__Clostridiales', 'f__Ruminococcaceae', 'g__Oscillospira
521642	k__Bacteria', 'p__Bacteroidetes', 'c__Bacteroidia', 'o__Bacteroidales', 'f__Bacteroidaceae', 'g__5-7N15
520385	k__Bacteria', 'p__Firmicutes', 'c__Clostridia', 'o__Clostridiales', 'f__Ruminococcaceae', 'g__
296424	k__Bacteria', 'p__Firmicutes', 'c__Clostridia', 'o__Clostridiales', 'f__Ruminococcaceae', 'g__Oscillospira
319662	k__Bacteria', 'p__Bacteroidetes', 'c__Bacteroidia', 'o__Bacteroidales', 'f__[Paraprevotellaceae]', 'g__CF231
289116	k__Bacteria', 'p__Firmicutes', 'c__Clostridia', 'o__Clostridiales
186092	k__Bacteria', 'p__Firmicutes', 'c__Clostridia', 'o__Clostridiales', 'f__Clostridiaceae', 'g__
302013	k__Bacteria', 'p__Bacteroidetes', 'c__Bacteroidia', 'o__Bacteroidales', 'f__[Paraprevotellaceae]', 'g__CF231
326382	k__Bacteria', 'p__Bacteroidetes', 'c__Bacteroidia', 'o__Bacteroidales', 'f__Bacteroidaceae', 'g__5-7N15
305099	k__Bacteria', 'p__Bacteroidetes', 'c__Bacteroidia', 'o__Bacteroidales', 'f__RF16', 'g__
341322	k__Bacteria', 'p__Firmicutes', 'c__Bacilli', 'o__Turicibacterales', 'f__Turicibacteraceae', 'g__Turicibacter
321610	k__Bacteria', 'p__Firmicutes', 'c__Clostridia', 'o__Clostridiales', 'f__Lachnospiraceae', 'g__Dorea
334216	k__Bacteria', 'p__Firmicutes', 'c__Clostridia', 'o__Clostridiales', 'f__Ruminococcaceae', 'g__
314304	k__Bacteria', 'p__Firmicutes', 'c__Clostridia', 'o__Clostridiales', 'f__Lachnospiraceae']
304482	k__Bacteria', 'p__Bacteroidetes', 'c__Bacteroidia', 'o__Bacteroidales', 'f__[Paraprevotellaceae]', 'g__CF231
287875	k__Bacteria', 'p__Firmicutes', 'c__Clostridia', 'o__Clostridiales', 'f__Ruminococcaceae', 'g__
308502	k__Bacteria', 'p__Firmicutes', 'c__Clostridia', 'o__Clostridiales', 'f__Peptostreptococcaceae', 'g__
568410	k__Bacteria', 'p__Bacteroidetes', 'c__Bacteroidia', 'o__Bacteroidales', 'f__Bacteroidaceae', 'g__5-7N15
590980	k__Bacteria', 'p__Firmicutes', 'c__Clostridia', 'o__Clostridiales', 'f__Ruminococcaceae', 'g__Oscillospira
517048	k__Bacteria', 'p__Firmicutes', 'c__Clostridia', 'o__Clostridiales', 'f__Ruminococcaceae', 'g__
4317034	k__Bacteria', 'p__Bacteroidetes', 'c__Bacteroidia', 'o__Bacteroidales', 'f__Bacteroidaceae', 'g__5-7N15
314328	k__Bacteria', 'p__Bacteroidetes', 'c__Bacteroidia', 'o__Bacteroidales', 'f__Bacteroidaceae', 'g__5-7N15
518013	k__Bacteria', 'p__Bacteroidetes', 'c__Bacteroidia', 'o__Bacteroidales', 'f__Bacteroidaceae', 'g__5-7N15
4306002	k__Bacteria', 'p__Bacteroidetes', 'c__Bacteroidia', 'o__Bacteroidales
319354	k__Bacteria', 'p__Bacteroidetes', 'c__Bacteroidia', 'o__Bacteroidales', 'f__Bacteroidaceae', 'g__5-7N15
317802	k__Bacteria', 'p__Bacteroidetes', 'c__Bacteroidia', 'o__Bacteroidales', 'f__Bacteroidaceae', 'g__5-7N15
323794	k__Bacteria', 'p__Firmicutes', 'c__Clostridia', 'o__Clostridiales', 'f__Lachnospiraceae', 'g__
528782	k__Bacteria', 'p__Firmicutes', 'c__Clostridia', 'o__Clostridiales', 'f__Ruminococcaceae', 'g__
4415144	k__Bacteria', 'p__Bacteroidetes', 'c__Bacteroidia', 'o__Bacteroidales', 'f__Bacteroidaceae', 'g__5-7N15
4311539	k__Bacteria', 'p__Bacteroidetes', 'c__Bacteroidia', 'o__Bacteroidales', 'f__S24-7', 'g__

New.CleanUp.ReferenceOTU39	k__Bacteria', 'p__Bacteroidetes', 'c__Bacteroidia', 'o__Bacteroidales', 'f__Bacteroidaceae', 'g__5-7N15
New.CleanUp.ReferenceOTU14308	k__Bacteria', 'p__Bacteroidetes', 'c__Bacteroidia', 'o__Bacteroidales', 'f__Rikenellaceae', 'g__
New.CleanUp.ReferenceOTU199	k__Bacteria', 'p__Firmicutes', 'c__Clostridia', 'o__Clostridiales', 'f__Ruminococcaceae', 'g__
New.CleanUp.ReferenceOTU7	k__Bacteria', 'p__Bacteroidetes', 'c__Bacteroidia', 'o__Bacteroidales
New.CleanUp.ReferenceOTU236	k__Bacteria', 'p__Bacteroidetes', 'c__Bacteroidia', 'o__Bacteroidales', 'f__Bacteroidaceae', 'g__5-7N15

Rectum n=9

297143	k__Bacteria', 'p__Firmicutes', 'c__Clostridia', 'o__Clostridiales
330414	k__Bacteria', 'p__Firmicutes', 'c__Clostridia', 'o__Clostridiales', 'f__Lachnospiraceae
526676	k__Bacteria', 'p__Bacteroidetes', 'c__Bacteroidia', 'o__Bacteroidales', 'f__S24-7', 'g__
304568	k__Bacteria', 'p__Firmicutes', 'c__Clostridia', 'o__Clostridiales', 'f__Lachnospiraceae']
297769	k__Bacteria', 'p__Bacteroidetes', 'c__Bacteroidia', 'o__Bacteroidales', 'f__Bacteroidaceae', 'g__5-7N15
524111	k__Bacteria', 'p__Firmicutes', 'c__Clostridia', 'o__Clostridiales', 'f__Ruminococcaceae', 'g__
355912	k__Bacteria', 'p__Firmicutes', 'c__Clostridia', 'o__Clostridiales', 'f__Clostridiaceae', 'g__Clostridium
529083	k__Bacteria', 'p__Firmicutes', 'c__Clostridia', 'o__Clostridiales', 'f__Ruminococcaceae', 'g__
584083	k__Bacteria', 'p__Firmicutes', 'c__Clostridia', 'o__Clostridiales
813277	k__Bacteria', 'p__Firmicutes', 'c__Clostridia', 'o__Clostridiales', 'f__Ruminococcaceae', 'g__
302013	k__Bacteria', 'p__Bacteroidetes', 'c__Bacteroidia', 'o__Bacteroidales', 'f__[Paraprevotellaceae]', 'g__CF231
326382	k__Bacteria', 'p__Bacteroidetes', 'c__Bacteroidia', 'o__Bacteroidales', 'f__Bacteroidaceae', 'g__5-7N15
334216	k__Bacteria', 'p__Firmicutes', 'c__Clostridia', 'o__Clostridiales', 'f__Ruminococcaceae', 'g__
525024	k__Bacteria', 'p__Firmicutes', 'c__Clostridia', 'o__Clostridiales', 'f__Clostridiaceae', 'g__
314304	k__Bacteria', 'p__Firmicutes', 'c__Clostridia', 'o__Clostridiales', 'f__Lachnospiraceae']
531310	k__Bacteria', 'p__Bacteroidetes', 'c__Bacteroidia', 'o__Bacteroidales', 'f__Bacteroidaceae', 'g__5-7N15
304482	k__Bacteria', 'p__Bacteroidetes', 'c__Bacteroidia', 'o__Bacteroidales', 'f__[Paraprevotellaceae]', 'g__CF231
308502	k__Bacteria', 'p__Firmicutes', 'c__Clostridia', 'o__Clostridiales', 'f__Peptostreptococcaceae', 'g__
518013	k__Bacteria', 'p__Bacteroidetes', 'c__Bacteroidia', 'o__Bacteroidales', 'f__Bacteroidaceae', 'g__5-7N15
319354	k__Bacteria', 'p__Bacteroidetes', 'c__Bacteroidia', 'o__Bacteroidales', 'f__Bacteroidaceae', 'g__5-7N15
323794	k__Bacteria', 'p__Firmicutes', 'c__Clostridia', 'o__Clostridiales', 'f__Lachnospiraceae', 'g__
528782	k__Bacteria', 'p__Firmicutes', 'c__Clostridia', 'o__Clostridiales', 'f__Ruminococcaceae', 'g__
4415144	k__Bacteria', 'p__Bacteroidetes', 'c__Bacteroidia', 'o__Bacteroidales', 'f__Bacteroidaceae', 'g__5-7N15
293937	k__Bacteria', 'p__Firmicutes', 'c__Clostridia', 'o__Clostridiales', 'f__Clostridiaceae', 'g__Clostridium

289992	k__Bacteria', 'p__Firmicutes', 'c__Clostridia', 'o__Clostridiales']
4311539	k__Bacteria', 'p__Bacteroidetes', 'c__Bacteroidia', 'o__Bacteroidales', 'f__S24-7', 'g__
