# Supplementary material for: Changes in bacterial community composition of Escherichia coli O157:H7 super-shedder cattle occur in the lower intestine
Source: PLoS One. 2017 Jan 31;12(1):e0170050. doi: 10.1371/journal.pone.0170050 (PMC5283656; doi:10.1371/journal.pone.0170050)
Supplement: S2 File — Fig A in S2 File. Differentially abundant genera in each gastrointestinal section as assessed using LEfSE. Fig B in S2 File. PCoA plot of the weighted UniFrac distances for lower and upper gastrointestinal samples. Fig C (A) in S2 File. PCoA plots of the weighted UniFrac distances for each gastrointestinal section. Fig C (B) in S2 File. PCoA plots of the weighted UniFrac distances for each animal for lower GI samples only (PDF) [file pone.0170050.s002.pdf]

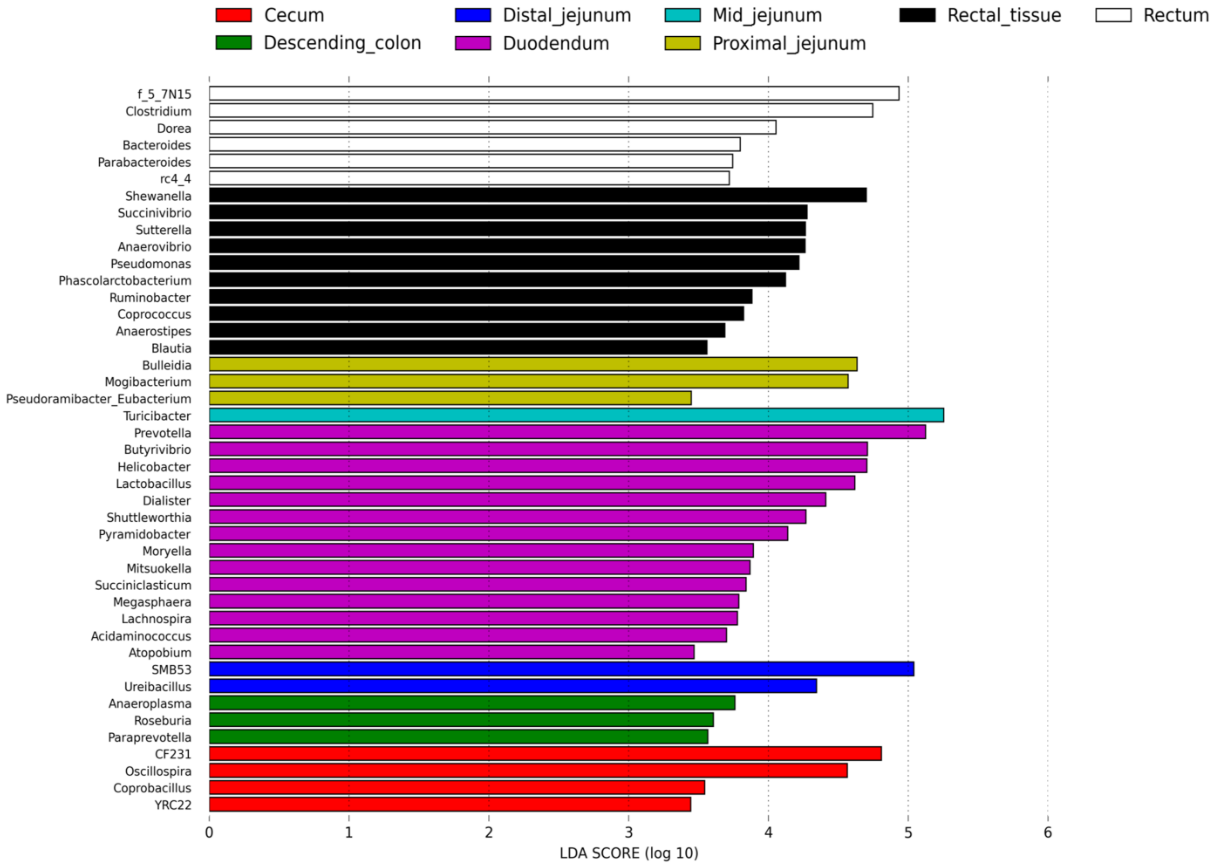

Fig. A. Differentially abundant genera in each gastrointestinal section as assessed using LEfSE. Only genera with a LDA score > 3.0 are displayed (n=10).

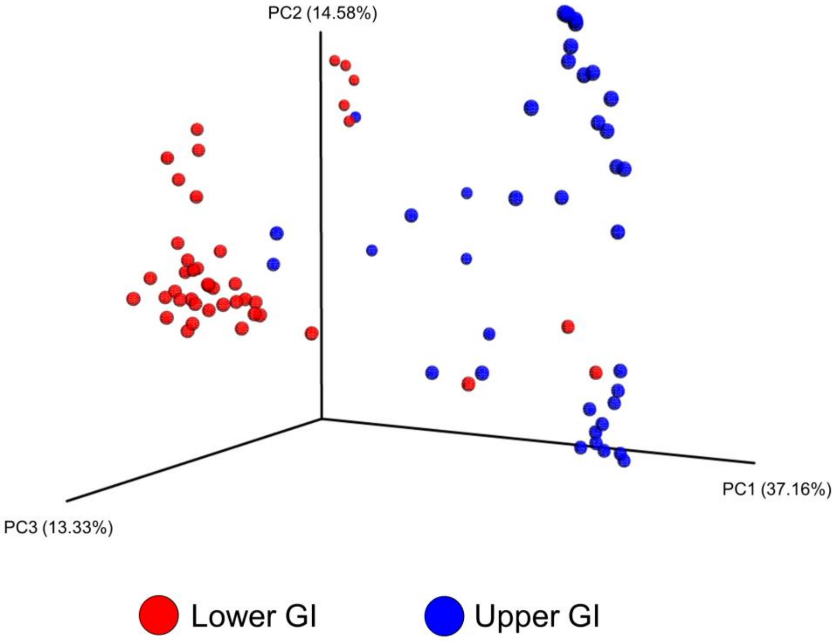

Fig. B. PCoA plot of the weighted UniFrac distances for lower and upper gastrointestinal samples. The percent variation explained by the principal coordinates is indicated on the axes.

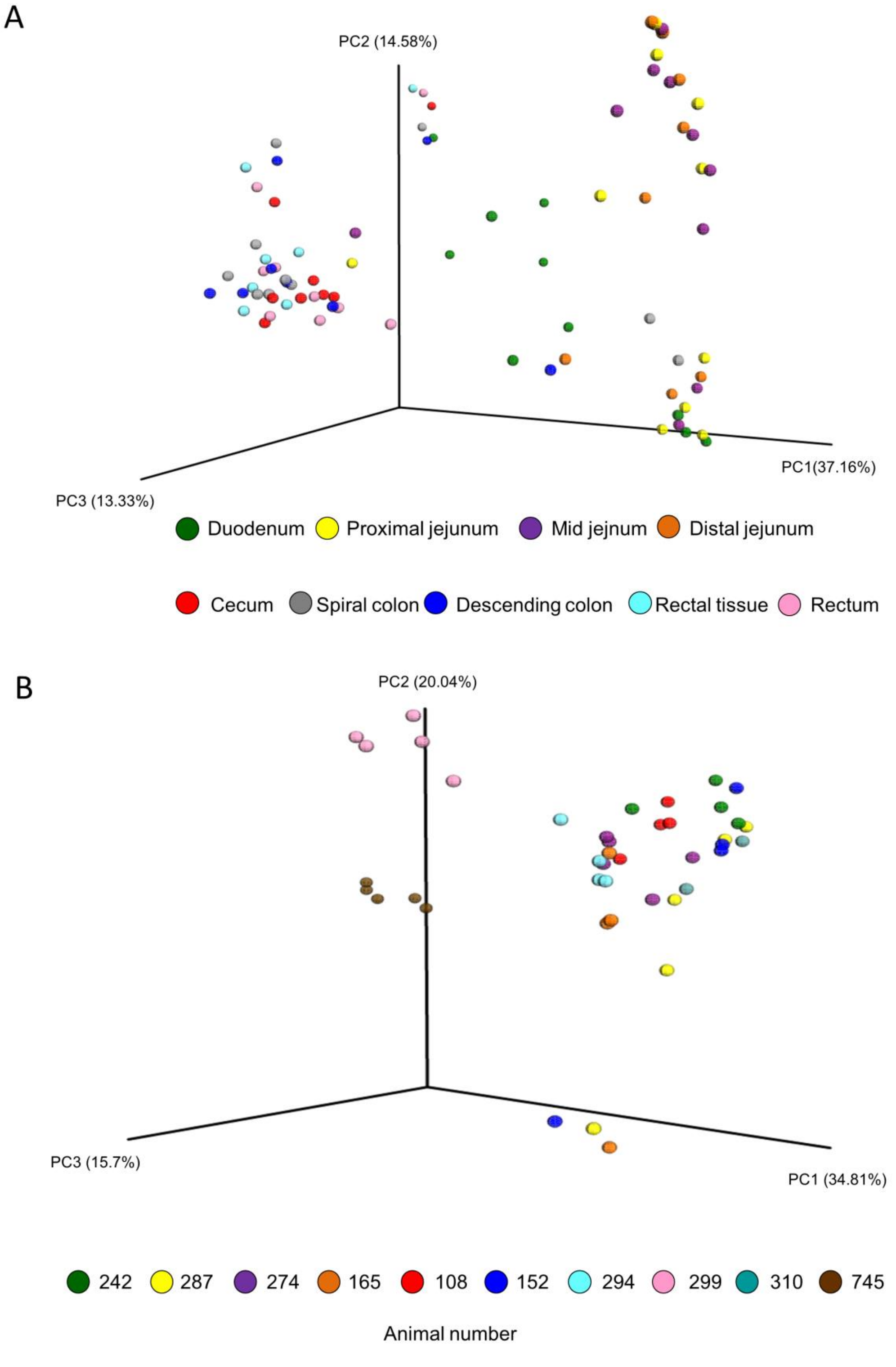
A
PC2 (14.58%)
PC1(37.16%)
PC3 (13.33%)
Duodenum
Proximal jejunum
Mid jejnum
Distal jejunum
Cecum
Spiral colon
Descending colon
Rectal tissue
Rectum
B
PC2 (20.04%)
PC3 (15.7%)
PC1 (34.81%)
242
287
274
165
108
152
294
299
310
745
Animal number

Fig. C. PCoA plots of the weighted UniFrac distances for A) each gastrointestinal section and B) animal for lower GI samples only. The percent variation explained by the principal coordinates is indicated on the axes.
